# Address of the Retiring President

**Published:** 1907-01

**Authors:** E. Bates Block

**Affiliations:** Atlanta, Ga.


### ADDRESS OF THE RETIRING PRESIDENT.

Delivered before the Fulton County Medical Society,
December 20, 1906.

By E. BATES BLOCK, M. D.

Gentlemen—It was my intention to address you to-night upon some medical subject, but there are some matters of general interest and importance which should be brought before you, and I could find no better time.

From a medical standpoint, our year has been a very successful one. Our printed program contained the titles of eighty-two papers, and three more were added during the year. As far as could be ascertained, twenty-nine papers failed to be read. There were four cases reported, and twelve cases or specimens exhibited, to the society, making practically seventy-two papers read during the year, or three at each meeting.

It seems to me that not enough time was devoted during the year to the general management of this society. Owing to the lateness of the time when the papers were finished, many of the members left before the general business of the society was considered, and this was usually hurried through with, in order to adjourn. I would suggest that a time limit be placed upon the medical portion of the meeting in order to leave more time for eneral business. If the meetings could start promptly at eight o'clock, this could be easily limited to ten o'clock.

While this society is rightly intended for the advancement of our medical knowledge, we owe it to the public and to our fellow practitioners to use our efforts to regulate the legitimate practice of medicine. We owe it to ourselves to protect ourselves against the impositions of our patients. There are, as you all know, people who go from one doctor to another, changing as often as a doctor tries to collect his fees, and never pay any of them. It is right that we should know these people and know how to deal with them. None of us would neglect to treat a charity case properly, but we have a right to know which case is to be regarded as a charity case. As you know, patients often send for us when there is no necessity for it, and even insist upon the visit after being told that it is unnecessary. If people are able and willing to pay their bills, and choose to spend their money in this way, they surely have a right to do so. But patients who have no intention of paying their bills have no right to wear a doctor out with unnecessary visits, many of which are forced upon the doctor at night, because the additional cost of a night visit can make no difference if the bill is not to be paid at all.

Many of them demand several visits a day, and could be better cared for in a charity hospital. By the co-operation of the members of our society it would be a very easy matter to have a complete index file of all of the patients who consult our members and fail to pay their bills. We have a right to this information, and it would enable us to save both time and expense and much needed rest, without injustice to our charity patients. With a slight increace in our dues, this record can be kept and will save our members many times what it cost them. A credit society here claims to have sixty-two doctors on its list of subscribers at twenty-five dollars each, making a total of fifteen hundred and fifty dollars subscribed by the medical profession, from which only a limited number derive benefit. If such an agency were conducted by our society, it could be conducted by a stenographer at three hundred dollars a year and the cost of stationary and a telephone. I must urge you to take this matter under consideration.

Nothing could be more surprising than the fact that in spite of the intellect and learning of the medical profession they have made no effort to utilize the knowledge that has been turned to account by the uncultured artisans of the world. There exist, as we know, almost as many kinds of labor unions as there are different kinds of labor, and in every instance it has resulted in an increase of wages and shorter working hours, when without them the great organizations of capital would have caused a decrease of wages, and have maintained the working hours. Surely if these men with families to support and a very meagle salary to support them on can afford to pay a portion of each month's wages to their union, we who are supposed to know more might follow in their footsteps, especially as we know the results they have attained. We all desire an improvement in the condition of the medical profession. But it seems that we are not willing to pay for it, either in time or money. Unless we given one or the other, we can expect nothing. It is necessary for us to engage a lawyer to represent our interests, to report un-licensed practitioners to the grand jury, to prosecute people who are practicing medicine without ever having attended a medical college, to have laws passed to protect legitimate practitioners. Does it not seem wrong to you that the State should require us to pass an examination and pay a yearly tax when it allows others to do just what we are doing and exacts nothing of them? Are the laws so framed that they are of any service if an unlicensed practitioner is brought before the court? Even the plumbers have protected themselves by their union, so that it is against the law for you to put a nut, a screw, or a washer on a pipe in your own house. No plumber can do the work without a license. Surely we have more intelligence than plumbers, but have we less power to utilize our intelligence. It is not a difficult matter for us to control the practice of medicine. Money is all that is necessary, and this we must have. Abolishing quackery, and the "medicine companies" practicing medicine, will not benefit the doctors who already have as much as they can do. It will benefit the doctors who are not making a living more than anyone else. Those who feel that they can least afford to help pay for this are the very ones who can least afford not to pay.

Let us see what the barber's union is doing in the United States and Canada. At their meeting two years ago, they reported in their treasury eighty-five thousand dollars, and it is now estimated to amount to about one hundred thousand dollars. Each barber pays into this union sixty cents a month, or seven dollars and twenty cents a year. Are not the physicians of Atlanta able to pay for their protection as much as barbers? I propose that a fund be established for this purpose, and that the dues to this society be increased to ten dollars per year.

I believe that probably one-third of the practice of medicine is illegitimate, though not according to the present laws, illegal. (I include here counter prescribing). If this be a fair estimate, it would increase the income of each legitimate practitioner one-third. Even if it be exaggerated ten-fold, on a basis of earning one hundred dollars a month, it will increase the income of each practitioner forty dollars per year, if we establish this fund. Owing to the recent interest in these matters excited by *Collier's Weekly*, the *Ladies' Home Journal*, and other papers, there is probably no time so ripe as the present for undertaking the accomplishment of this object.

You are probably all aware of the difficulties arising in many of our Georgia towns on account of the life insurance companies reducing the fees for examination from five dollars to three dollars. We have here an example of capital against labor, which we mus tmeet by union or submit to individually, and be prepared for still further reductions in the future. Unless we establish a fixed fee for such examinations and adhere to it, the insurance companies may naturally be expected to reduce their fees so low that it will cease to be profitable to any educated physician. While our present examiners, including myself, may regard it a hardship to have our society distate the fee it shall charge for examination, and make its adherence to it a requisite for his continued membership in the society, the time will come when the medical profession will profit by it. I, for one, will be glad to have this law adopted and will adhere to it.

To sum up these matters for your consideration and, if you think well of it, for your adoption, I propose:

1. That the dues be increased to ten dollars a year.
2. hTat a fund be created for the establishment of proper laws, for the enforcement of these laws, and the suppression of illegitimate practice.
3. That a portion of the dues be applied to the keeping of reports upon the financial responsibility of patients.
4. That the life insurance fees be made five dollars, and that no member of the society shall accept less, making adherence to this law a requisite of his continued membership in the society.
5. That the medical portions of our meetings be limited to an hour fixed by the society.

